# Educational Intervention to Improve Sexual Health and Quality of Life in Survivors of Breast and Gynecological Cancer: Protocol for a Mixed Methods Feasibility Study

**DOI:** 10.2196/80567

**Published:** 2026-02-27

**Authors:** Nathalia Salazar Falla, María de los Ángeles Navas Jojoa, Juan David Lalinde Riviño, Auramaría Escandón Bucheli, Juan Sebastián Galindo-Sánchez

**Affiliations:** 1 Department of Internal Medicine, Fundación Valle del Lili, Cra 98 No. 18 - 49, Cali, Valle del Cauca, 760032, Colombia, 57 6023319090 ext 8364; 2 Facultad de Ciencias de la Salud, Universidad Icesi, Cali, Valle del Cauca, Colombia; 3 Department of Gynecology, Consorcio Clínica Nueva Rafael Uribe Uribe, Cali, Valle del Cauca, Colombia; 4 Department of Gynecologic Oncology, Fundación Valle del Lili, Cali, Valle del Cauca, Colombia; 5 Department of Psychiatry, Fundación Valle del Lili, Cali, Valle del Cauca, Colombia; 6 Centro de Investigaciones Clínicas, Fundación Valle del Lili, Cali, Valle del Cauca, Colombia

**Keywords:** female genital neoplasms, breast neoplasms, survivors, health education, sexual health, quality of life

## Abstract

**Background:**

Sexual health is often underaddressed in cancer care, especially among survivors of breast and gynecological cancer. In Colombia, treatment side effects, cultural stigma, and limited training among health care providers affect well-being. Although international guidelines promote patient-centered and culturally sensitive approaches, few interventions have been implemented in low- and middle-income settings.

**Objective:**

This study aimed to describe the feasibility and acceptability of an educational psychosexual intervention for survivors of breast and gynecological cancer, including design, integration of qualitative and quantitative methods, core components, measurement time points, and a prespecified analysis plan.

**Methods:**

This is a prospective, single-group feasibility study at a university hospital in Colombia. Participants are adult women who completed treatment for breast or gynecological cancer at least 2 years before enrollment. The intervention combines tiered educational workshops, semistructured group interviews, and optional individualized counseling. Qualitative data will explore perceptions, barriers, and facilitators related to sexual health and well-being. Quantitative outcomes include the Female Sexual Function Index and European Organisation for Research and Treatment of Cancer Quality of Life Questionnaire Core 30 before and after the intervention to estimate change. Pre- and postintervention scores will be compared with paired tests and CIs. Qualitative data will undergo reflexive thematic analysis. Mixed findings will inform feasibility, acceptability, and practical recommendations. The intervention was co-designed with survivors and a multidisciplinary team, refined by expert review and a manual, and pilot-tested to optimize acceptability, logistics, and fidelity.

**Results:**

The study was funded in December 2024 and approved by the Comité de Ética en Investigación Biomédica of Fundación Valle del Lili. Recruitment began in March 2025 and is expected to end in September 2025. As of July 2025, 13 participants had enrolled (n=2, 15.4% survivors of breast cancer; n=11, 84.6% survivors of gynecological cancer). Based on pretrial piloting, shorter session blocks and optional hybrid delivery were made available to support adherence while preserving core content and objectives. Final analysis is planned for October 2025, with results expected in December 2025.

**Conclusions:**

This protocol evaluates the feasibility and acceptability of a codesigned, context-adapted psychosexual education model. Findings will guide implementation refinements and the design of a future comparative study of effectiveness and longer-term outcomes and may inform sustainable integration of sexual health into survivorship care in low- and middle-income settings. The model is intended to be replicable and scalable across oncology and other chronic disease contexts.

## Introduction

### Background

Among patients with gynecological cancer, research has traditionally focused on oncological treatments aimed at prolonging overall survival and progression-free survival. However, quality of life is increasingly recognized as crucial for women who survive these diseases. Sexual health is a fundamental component of overall well-being and quality of life, yet it remains one of the most overlooked aspects of oncological care, especially among women who have survived gynecological cancers. A systematic review by Sousa Rodrigues Guedes et al [1] reported that 30% to 80% of women with cancer experience sexual dysfunction and that the likelihood of this condition is 2.7 to 3.5 times higher than in women without cancer.

In gynecological cancers, where organs directly involved in sexual function may be affected, the burden of sexual dysfunction is particularly high. For example, Firmeza et al [2] reported a 5% to 16% prevalence of dyspareunia after laparoscopic radical hysterectomy, and Jensen et al [3] found that among patients with cervical cancer receiving radiotherapy, 55% reported mild to severe dyspareunia and 30% were dissatisfied with their sexual life. In Colombia, survivors face additional barriers to sexual well-being, including physical treatment effects, psychological distress, cultural taboos, and limited training among health care providers [4-7]. Across gynecological cancers, surgery, pelvic radiotherapy, chemotherapy, and endocrine therapies contribute to decreased desire and arousal, vaginal dryness, dyspareunia, pelvic floor pain, body image disturbance, and relationship strain; recent reviews highlight multifactorial mechanisms (anatomical, neurovascular, hormonal, and psychosocial) and underscore the need for proactive assessment during survivorship care [8].

Beyond anatomical or physiological measures, such as vaginal dilators or estrogen gels, the psychosexual component is central to women’s sexual pleasure and recovery. Trials report benefits of psychosocial approaches: telephone counseling improved depressive symptoms and gynecological or cancer-specific concerns [9], and a nurse-led sexual rehabilitation program improved sexual function from 1 to 12 months after treatment [10].

Building on this, brief, structured clinician- and internet-delivered interventions have improved sexual concerns and communication, including a clinician communication pilot [11], documented patient preferences for routine discussion [12], and web-based psychoeducational programs (Fex-Can) under randomized evaluation [13,14]. Counseling frameworks such as PLISSIT (permission, limited information, specific suggestion, intensive therapy) and BETTER (bring up, explain, tell, time, education, record) provide practical, stepwise scripts for time-limited visits [15] and show comparative advantages [16], although the evidence base specific to survivors of gynecological cancer remains limited [9,10].

Worldwide, organizations such as the World Health Organization (WHO) [17]; the United Nations Educational, Scientific, and Cultural Organization [18]; and the World Association for Sexual Health (WAS) [19] advocate for integrated, patient-centered approaches that recognize sexual health as a fundamental right and an essential element of postcancer recovery. Despite these calls, translation into routine practice remains challenging in many low- and middle-income countries, where issues of access and cultural relevance persist [17,18]. Moreover, the evidence base is derived largely from high-income settings [20], motivating context-specific, culturally adapted interventions such as the one evaluated in this study.

This study is primarily designed to evaluate the feasibility and acceptability of implementing an educational psychosexual intervention for survivors of breast and gynecological cancer. As a secondary objective, it will estimate the preliminary effectiveness of the intervention. It is important to note that this is not a definitive effectiveness trial but an initial step to determine whether the intervention can be successfully implemented and is acceptable to participants.

### Protocol Objectives

This protocol aims to describe the feasibility and acceptability of implementing EDUSEXONCO—an educational psychosexual intervention for survivors of breast and gynecological cancer—including its design, mixed methods integration, intervention components, measurement time points, and prespecified analysis plan.

### Prespecified Study Aims

The primary aim is to describe the feasibility and acceptability of implementing an educational strategy to promote sexual health and quality of life in a cohort of Colombian patients (EDUSEXONCO project).

The secondary aims are to (1) estimate within-group change in sexual function (Female Sexual Function Index; FSFI) and health-related quality of life (European Organisation for Research and Treatment of Cancer Quality of Life Questionnaire Core 30; EORTC QLQ-C30) from baseline (before session 1) to after the intervention (end of session 3) and (2) explore the relationship between quantitative changes in FSFI and EORTC QLQ-C30 scores and qualitative findings on sexual well-being, communication, agency, and body image from baseline (before session 1) to after the intervention (end of session 3).

## Methods

### Design

This is a prospective, single-center, single-group (pretest-posttest) mixed methods feasibility study designed to evaluate the implementation and acceptability of an educational psychosexual intervention for survivors of gynecological cancer. Estimating preliminary effectiveness is a secondary objective. This is not a definitive effectiveness trial but an initial step to determine whether the intervention can be delivered successfully and is acceptable to participants.

This study uses an embedded convergent mixed methods design, giving equal priority to quantitative and qualitative strands. Quantitative data (FSFI and EORTC QLQ-C30 scores) will be collected at baseline (before session 1) and after the intervention (end of session 3). Qualitative data (focus groups, semistructured interviews, participant observation field notes, and anonymous diaries) will be gathered after each session. Integration will occur during interpretation through meta-inferences and joint displays that juxtapose pretest-posttest quantitative changes with qualitative themes related to sexual well-being, communication, agency, and body image. We will seek convergence, complementarity, and expansion; discrepancies will be examined to refine inferences and inform practical recommendations.

### Recruitment

#### Overview

We will use consecutive sampling from survivorship and gynecological oncology outpatient clinics. Procedures include (1) weekly electronic medical record prescreening (diagnosis, treatment completion of ≥2 years, age of ≥18 years, and active follow-up), (2) clinician referral during routine visits via a standardized slip, (3) approach in the clinic or telephone or email contact within 7 days, (4) brief scripted eligibility confirmation, and (5) provision of written informed consent (in Spanish). A recruitment log (approached, eligible, provided consent, and ineligible; reasons for decline; and dates) will quantify feasibility. Retention supports include flexible scheduling, reminders 48 to 72 hours before sessions, and transportation provided for participants who require it (funded by the study grant and arranged by the research team in coordination with institutional services).

#### Recruitment Setting

The study will take place at Fundación Valle del Lili (FVL), a university-affiliated tertiary hospital in Cali, Colombia, recognized as a leading center for high-complexity care, including oncology services, in Latin America. FVL provides coverage across southwestern Colombia, serving a population with significant ethnic diversity, and maintains agreements with multiple health plans. Since 2017, the institution has operated a functional cancer unit dedicated to delivering comprehensive care for patients with oncological diseases. This unit offers facilities and procedures for initiation, follow-up, and monitoring of cancer treatment supported by an experienced multidisciplinary team [21]. The study will be conducted from 2024 to 2025 within this setting.

#### Study Population

Participants will be adult women (aged ≥18 years) who have survived breast or gynecological cancer (cervical, uterine, vaginal, vulvar, or ovarian), completed oncological treatment at least 2 years prior to enrollment, and remain under follow-up at FVL.

### Eligibility Criteria

The eligibility criteria are shown in Textbox 1.

Textbox 1. **Eligibility criteria.**

**Inclusion criteria**

- Women aged ≥18 years
- History of breast or gynecological cancer (cervical, uterine, vaginal, vulvar, or ovarian)
- Completion of primary oncological treatment ≥24 months before enrollment (ongoing endocrine therapy or topical vaginal estrogen is allowed)
- Being disease free or having stable disease not requiring urgent oncological treatment as per the most recent clinic note
- Active follow-up at Fundación Valle del Lili
- Being Spanish speaking and able to complete study procedures
- Ability and willingness to provide written informed consent and attend the 3 intervention sessions (in person or hybrid)

**Exclusion criteria**

- Cognitive impairment that prevents informed participation (clinician judgment or Mini-Cog <3 if uncertain)
- Medical or psychiatric instability that would compromise safe participation (eg, uncontrolled pain crisis, acute severe depression with suicidality, or psychosis) per the treating clinician
- Active cancer recurrence requiring immediate treatment at screening
- Any condition that, in the investigator’s judgment, precludes group participation (eg, major morbidity or infectious isolation requirements) or completion of study assessments
- Concurrent enrollment in another sexual health behavioral intervention study

A brief operational screening checklist is provided in Multimedia Appendix 1.

### Intervention

EDUSEXONCO is a structured psychoeducational program to enhance sexual health and quality of life in survivors of gynecological cancer.

The intervention targets modifiable psychosexual determinants—knowledge, communication, agency, body image, dyspareunia self-management, and pelvic floor function—through evidence-based education, brief skill practice, and optional individualized counseling to support recovery of sexual well-being.

Materials include a facilitator manual (objectives and scripts for sensitive topics), slides and handouts, 3D anatomical models, fidelity checklists, attendance and adherence logs, and brief take-home practice sheets. Delivery comprises three components: (1) large-group theoretical-practical workshops organized in 3 progressive levels (basic, intermediate, and advanced), (2) semistructured group interviews (6‐8 participants) to elicit lived experience and contextual barriers and facilitators, and (3) optional individual counseling when chosen. Brief skills (communication drills, breathing and relaxation, and pelvic floor basics) are embedded in each session.

EDUSEXONCO is delivered as a sequence of 3 progressive theoretical-practical workshop levels (basic, intermediate, and advanced) to support gradual learning and skill acquisition. Each level includes two complementary strands: (1) a medical-sexual strand focused on anatomy and sexual response, treatment-related sexual changes, symptom self-management (eg, lubrication, pain, and dyspareunia), and safe use of supportive resources; and (2) a sociopsychological strand focused on stigma reduction, communication, consent and boundaries, self-compassion and body image, emotional regulation, and social reintegration. Each workshop incorporates brief guided practice (eg, “anatomy tour” using 3D models, communication scripts, sensate focus–inspired exercises, paced breathing, pelvic floor downtraining, and gentle yoga and relaxation), a take-home task to reinforce learning between sessions, and fidelity markers (eg, coverage of key items, completion of role-plays or practice, and delivery of handouts) to standardize delivery across facilitators. The full-day schedule is provided in Multimedia Appendix 2. A detailed topic-by-level syllabus including objectives, key content, practices, materials, leads, take-home tasks, fidelity markers, and qualitative prompts is provided in Multimedia Appendix 3. Examples of the semistructured interview guide are provided in Multimedia Appendix 4.

In-person group delivery takes place in education or rehabilitation rooms with acoustic privacy at FVL comprising 3 sequential full-day sessions (approximately 8 hours each) over approximately 3 weeks (roughly 24 hours in total). The intervention includes optional home practice for 10 to 15 minutes per day.

Language and examples are culturally adapted to Colombia, alternatives are provided for participants without a partner, activities are adapted for pain or mobility limitations, and Spanish-language materials target approximately B1 to B2 readability.

Fidelity is monitored using per-session checklists covering objectives, core activities, materials, timing, inclusivity, skill practice, and end-of-session outputs (anonymous diaries). Attendance and adherence logs capture session-level participation (sessions 1‐3), completion of pretest and posttest questionnaires, participation in the group interview and optional 1:1 counseling, and take-home practice reporting.

Usual care is allowed (gynecology, psycho-oncology, pelvic floor therapy, and analgesia). Cointerventions (eg, topical estrogen, dilators, and sex therapy) are documented. Adverse events (eg, pelvic pain flare or marked emotional distress) are recorded and managed via institutional pathways; urgent cases are referred to psycho-oncology immediately.

A multidisciplinary team will deliver and evaluate the intervention. All facilitators will complete a 2-hour orientation on the facilitator manual, stigma-free and rights-based communication, trauma-informed practices, confidentiality, and use of fidelity tools (presession and per-session checklists). Interviewers will receive additional coaching on qualitative interviewing and reflexivity memoing. Each professional involved in the study will assume 2 primary roles: participation in the theoretical-practical workshops according to their field of expertise and moderation or support during semistructured interviews focusing on topics related to their professional area. The team comprises the following members:

- Principal investigator and study clinician (internist or gynecological oncologist)—oversees protocol fidelity and participant safety, addresses medical queries arising from workshops or interviews, and adjudicates eligibility or adverse event decisions as needed.
- Sexual health physician and sexologist—leads medical-sexual content on postcancer sexual physiology and function, pleasure, autoeroticism, and nonpharmacologic pain or dyspareunia strategies and provides brief one-to-one counseling when indicated.
- Psychologist—facilitates sessions on emotions, anxiety management, self-esteem, body image, and stigma; moderates group interviews; and manages emotional crises and referral to psycho-oncology per institutional pathways.
- Social worker—addresses sociocultural and socioeconomic barriers, family and social support networks, and reintegration and identifies cases requiring individualized follow-up or referral.
- Pelvic floor physical therapist—teaches basic pelvic floor contraction and relaxation, breathing, and posture for pain modulation; clarifies rehabilitation questions; and links exercises to sexual function.
- Nutritionist—provides guidance on nutrition to support recovery, energy, and quality of life; answers participant questions; and aligns advice with medical care.
- Yoga and relaxation instructor—leads brief, adapted breathing and posture practices to support emotional and physical well-being and offers alternatives for pain or mobility limitations.
- Sex educator—delivers comprehensive sexual education (communication skills, sexual aids, and diverse expressions of sexual health) using inclusive, culturally appropriate language.
- Data collection specialist and qualitative research assistant—manages audio recordings, field notes, deidentification, and secure transfer for transcription; maintains recruitment and fidelity logs; and supports postsession reflexivity memos.

### Role Boundaries and Safety

Companions may attend large-group workshops when desired by the participant but do not join interviews or individual counseling. Only trained team members deliver content in their scope; urgent clinical or emotional issues are escalated to the study clinician and psycho-oncology. All adverse events are documented and handled per institutional policy.

### Measures and Outcomes

The primary objective is feasibility and acceptability. We will quantify feasibility (eligibility, invitation, consent, recruitment rate, retention, attendance and adherence, fidelity, and data completeness) and acceptability (postsession satisfaction, perceived usefulness, and comfort; recommendation item scored from 0‐10). A priori thresholds are as follows: retention of ≥80%, attendance of ≥80% per session, fidelity of ≥80%, data completeness of ≥85%, no serious adverse events, acceptability mean of ≥4.0/5, and net promoter score of ≥30.

As a secondary objective, this study assesses quantitative outcomes using validated psychometric tools and qualitative dimensions using reflexive thematic analysis. Mixed methods integration will offer a comprehensive understanding of EDUSEXONCO’s effects on sexual rehabilitation and psychosocial recovery.

### Quantitative Component

#### Sexual Function

The FSFI is a validated 19-item instrument that assesses 6 domains of sexual function: desire, arousal, lubrication, orgasm, satisfaction, and pain. Each domain generates an individual score, and the total FSFI score is the sum of all domain scores, with a maximum of 36 points. A total score below 26.55 indicates sexual dysfunction. Authorization for the use of the FSFI has been obtained [22].

#### Health-Related Quality of Life

The EORTC QLQ-C30 version 3.0 is a validated 30-item instrument designed to assess health-related quality of life across 3 scales: functional, global quality of life, and symptoms. The functional and global scales range from 0 to 100, with higher scores indicating better functioning or overall quality of life. The symptom scale (eg, fatigue, pain, and insomnia) also ranges from 0 to 100, where lower scores reflect fewer symptoms and, therefore, better quality of life. Authorization for the use of the EORTC QLQ-C30 has been obtained [23].

### Qualitative Component

Qualitative insights on barriers, facilitators, and perceptions of sexual health and well-being will be derived from focus groups, semistructured interviews, participant observation field notes, and anonymous postsession diaries.

### Sociodemographic and Clinical Characteristics

At session 1 (before the intervention), participants will complete a brief self-administered questionnaire capturing age, educational level, employment status, relationship status, parity, menopausal status, time since diagnosis and completion of primary treatment, cancer type and stage (self-reported), prior and current treatments (including topical vaginal estrogen), relevant comorbidities and medications, pelvic floor therapy history, and sexual activity and partner status. Sensitive items are optional and include a “prefer not to answer” choice.

Participants will complete the sociodemographic questionnaire and baseline FSFI and EORTC QLQ-C30 at session 1 and repeat the FSFI and EORTC QLQ-C30 at session 3. Focus groups and semistructured interviews will be audio recorded and transcribed verbatim; participant observation field notes and anonymous diaries will be collected after each session. Optional one-to-one counseling is not recorded; clinicians may keep brief field notes. Trained staff will enter data into REDCap (Research Electronic Data Capture; Vanderbilt University).

Before each in-person session, participants receive a telephone call inviting them to attend the session. During the call, transportation needs are assessed; if necessary, transportation is arranged by the research team. Participants are informed that meals and refreshments are provided free of charge. If desired, participants may bring an accompanying adult family member who may attend the group sessions but will not participate in interviews or individual counseling to maintain privacy and confidentiality. To uphold participants’ autonomy and freedom, all individuals are explicitly informed that voluntary withdrawal is allowed at any time without penalty. This ensures full participant control over involvement and minimizes the risk of perceived coercion.

Personal, religious, or moral values will not be imposed. No judgments about patients’ sexual lives that could generate stigma, guilt, or shame are permitted. Inappropriate or nonconsensual language or materials are prohibited. Explicit or mocking sexual content is prohibited unless educationally justified and previously consented to by participants. Private information will not be disclosed without authorization. It will be strictly forbidden to deny or ignore the needs of lesbian, gay, bisexual, transgender, and queer survivors. Heteronormative assumptions are not permitted; all content must be inclusive and adapted to participants with nonbinary identities or same-sex partners. Unqualified or clinically unsupported interventions will not be allowed. All professionals must present credentials accrediting their expertise and complete training in cultural sensitivity and sexual ethics to prevent stigma and bias.

### Sample Size

The quantitative pretest-posttest sample size was calculated based on mean differences and an adjusted SD of 0.69, informed by prior studies such as Starting the Conversation, a pilot trial using the FSFI to assess sexual function in survivors of gynecological cancer [11]. Assuming a moderate effect size (*d*=0.5), a 95% confidence level, and 80% power, the estimated sample size is 60 participants. Calculations were performed using G*Power (Heinrich Heine University Düsseldorf), a standard tool for sample size estimation in mean difference studies.

For the qualitative strand, sample adequacy is guided by the information power principle—considering study aim, sample specificity, quality of dialogue, and analytic strategy—rather than numeric saturation targets [24]. We purposefully seek heterogeneity in survivorship stage and symptom profiles to capture a range of experiences relevant to sexual health education.

### Analysis Plan

#### Quantitative Analysis

Continuous variables will be summarized as means with SDs or medians with IQRs depending on the distribution, assessed using the Shapiro-Wilk test. Categorical variables will be presented as absolute and relative frequencies. Primary analyses will evaluate within-group changes (session 1 vs session 3) in FSFI and EORTC QLQ-C30 scores. For continuous outcomes, paired 2-tailed *t* tests will be used when normality assumptions are met; otherwise, Wilcoxon signed rank tests will be applied. A 2-sided significance level of α=.05 will be adopted. Effect sizes will be reported (Cohen *dz* for paired *t* tests; *r* for the Wilcoxon signed rank test), along with 95% CIs and graphical representations of response distributions. Missing data will be described; if they constitute >5% and are plausibly missing at random, an exploratory sensitivity analysis using multiple imputation will be performed.

#### Qualitative Analysis

Reflexive thematic analysis will be used following the framework by Braun and Clarke [25], prioritizing researcher reflexivity and active theme development over codebook reliability metrics. The analytic process will include (1) familiarization with transcripts and field notes, (2) inductive semantic and latent coding, (3) initial theme generation, (4) theme review against extracts and the full dataset, (5) theme definition and naming, and (6) construction of an analytic narrative linking themes to research questions and quantitative findings. Two researchers will independently code an initial subset to capture diverse interpretations, followed by reflexive dialogue. Subsequent coding will be led by the primary analyst with iterative peer debriefs focused on interpretive rigor rather than forced consensus. Reflexivity memos and an audit trail (decision logs and code or theme evolution) will be maintained. Credibility will be supported through data triangulation and thick description, dependability and confirmability will be supported through the audit trail, and transferability will be supported through detailed contextual reporting.

#### Reflexivity and Trustworthiness

The qualitative team (clinicians and educators experienced in sexual health and oncology) will maintain a reflexive stance through positionality statements, analytic memos, and documented decision trails. Credibility will be obtained via prolonged engagement across sessions, reflexive peer debriefs, and participant “member reflections” focused on emergent interpretations (not transcript verification). Dependability will be ensured based on an audit trail (versioned codebooks, memos, and decision logs) and clear documentation of analytic moves. Confirmability will be derived from reflexive journaling and an evidentiary chain linking raw data to themes and interpretations. Transferability will be dependent on “thick” descriptions of the setting, participants, and delivery to support readers’ judgments of applicability to similar contexts.

#### Mixed Methods Integration

Integration will occur at the interpretation stage through meta-inferences and joint displays juxtaposing quantitative pretest-posttest changes (FSFI and EORTC QLQ-C30 scores) with qualitative themes (eg, sexual well-being, communication, agency, and body image). We will seek convergence, complementarity, and expansion; discrepancies will be explicitly examined to refine interpretations and inform practical recommendations.

### Pilot Test

EDUSEXONCO was developed through a structured, user-centered process with three phases: (1) formative co-design, (2) content finalization and manualization, and (3) pretrial piloting and feasibility testing.

### Formative Co-Design

We held participatory workshops with survivors (breast and gynecological cancer), clinicians (gynecological oncology, internal medicine, and psychiatry), and allied professionals (psychology, social work, pelvic floor physiotherapy, nutrition, yoga and relaxation, and sexual education). Workshops identified priority needs, culturally appropriate language, and acceptable delivery formats. Preliminary materials (slides, handouts, and 3D educational models) were iteratively refined using survivor feedback and brief “think-aloud” walk-throughs to optimize clarity, tone, and inclusiveness.

### Patient and Public Involvement

Survivors of breast and gynecological cancers contributed to co-design (prioritizing topics, refining language, shaping levels, and informing 3D educational models) and will continue to provide iterative feedback on content and delivery during the study. They will also be invited to participate in dissemination and the codevelopment of implementation recommendations for scale-up.

#### Content Finalization and Manualization

A multidisciplinary panel reviewed all modules against international guidance (WHO; United Nations Educational, Scientific, and Cultural Organization; and WAS) and local practice to ensure accuracy, cultural relevance, and inclusivity. We developed a facilitator manual (learning objectives, session flow, time allocations, materials, and scripts for sensitive topics); fidelity tools (checklists and attendance and adherence trackers); and brief staff training on stigma-free, rights-based communication. Minor wording and sequencing changes enhanced accessibility for diverse literacy levels.

#### Pretrial Piloting and Feasibility Testing

Core components were piloted with a convenience sample of eligible survivors (n=15) to assess feasibility (recruitment and retention, session length, and logistics), acceptability (satisfaction, perceived usefulness, and comfort discussing sexual health), and fidelity (adherence to the manual). During pretrial piloting, full-day sessions were identified as a potential feasibility barrier; consequently, contingency adaptations (shorter session blocks, optional hybrid delivery, and flexible scheduling) were prepared while preserving the protocol’s objectives and core content. In this study, brief participant and facilitator feedback will be reviewed after each session to inform minor, prespecified logistical refinements (eg, scheduling and session length and pace) for subsequent groups without modifying core content or learning objectives. These findings guided the final structure of the sessions and the implementation plan to be used.

### Ethical Considerations

The protocol was approved by the Research Ethics Committee of FVL (protocol 2024.248). All participants will provide written informed consent before enrollment. The informed consent form will outline study activities and the use of data for analyses aligned with the study objectives. Participation is voluntary, and patients may decline without any impact on the provision of usual clinical care. Participants will not receive financial compensation or additional services; however, the cost of transportation from their residence to FVL and a meal voucher will be covered.

Only trained team members will deliver content within their scope of practice. Any clinical, psychological, or emotional issues arising from the implementation of the EDUSEXONCO intervention are escalated to the study clinician and the psycho-oncology team, who are professionally qualified to manage such situations. All adverse events are documented and handled according to the institutional policy of FVL. If necessary, participants may be referred to the institution’s emergency department, accompanied by the psycho-oncologist or a study representative, to initiate the required standard clinical care.

Ethical safeguards include respect for autonomy, confidentiality, and the right to withdraw at any time without affecting clinical care. Procedures are in place to minimize risk when addressing sensitive topics, including trauma-informed facilitation, private spaces for optional one-on-one counseling, and rapid referral pathways to psycho-oncology when indicated. All data will be deidentified and stored in secure, password-protected systems accessible only to authorized team members. Audio files and transcripts will be stored on secure institutional servers at the Clinical Research Center of FVL; identifiers will be removed during transcription, and a linkage file will be kept separately under restricted access. Paper materials (consent forms and checklists) will be stored in locked cabinets within restricted-access offices. Data retention will comply with institutional and national regulations. No photographs or video recordings of participants will be taken.

### Dissemination and Implementation Plan

In line with open science and transparency principles, we plan to disseminate study materials and outputs through (1) open access educational resources (eg, facilitator guides and modules) where appropriate, (2) interdisciplinary training workshops for health care providers to support culturally competent sexual health counseling in survivorship care, (3) presentations at relevant national and international scientific meetings and patient support networks, and (4) partnerships with academic and community stakeholders to support integration into clinical education and survivorship pathways. These activities aim to facilitate future replication and support sustainable incorporation of sexual health education into oncology survivorship care.

This protocol describes the rationale, intervention components, and mixed methods feasibility and acceptability evaluation of EDUSEXONCO, a co-designed, context-adapted educational psychosexual program for survivors of breast and gynecological cancer in Colombia. This study will assess implementation outcomes—including recruitment, retention, session attendance, fidelity to the manualized content, and completeness of quantitative and qualitative data collection—alongside participants’ acceptability and perceived usefulness of the intervention.

The findings will inform refinement of the EDUSEXONCO curriculum and delivery format (including prespecified contingency options) and will provide context-specific implementation knowledge to support integration of sexual health education into survivorship care pathways in low- and middle-income settings. Results from this feasibility study will also guide the design of a subsequent comparative, multisite study with longer follow-up to evaluate effectiveness and scalability.

## Results

This study is funded by the Women’s World Banking Colombia Foundation, awarded in December 2024, and received ethics approval from FVL. Recruitment began in March 2025 and is planned until September 2025. As of July 2025, a total of 13 participants have been enrolled (n=2, 15.4% with breast cancer and n=11, 84.6% with gynecological cancer). During feasibility activities, full-day sessions were identified as a potential barrier to attendance; accordingly, contingency adaptations (shorter session blocks and optional hybrid delivery) were suggested and will be considered as needed without altering protocol objectives or core content. Quantitative and qualitative data analysis is scheduled for October 2025; final results are expected by December 2025.

## Discussion

### Anticipated Findings and Contribution

This feasibility and acceptability study is designed to determine whether a co-designed, multidisciplinary educational psychosexual intervention (EDUSEXONCO) can be delivered as planned and is acceptable to survivors of gynecological and breast cancer in a Colombian university hospital setting. We anticipate generating actionable implementation evidence on recruitment and retention, session attendance, fidelity to the manualized content, and completeness of quantitative and qualitative data collection. In addition, we expect to identify participants’ perceived usefulness of the sessions, barriers to participation, and opportunities to refine content and delivery to better address sexual health needs during survivorship care.

### Comparison With Prior Work

Prior research has consistently documented that sexual health concerns are common among survivors of cancer and that health care professionals frequently report limited training, time constraints, and discomfort in addressing sexuality in routine oncology care [12,26]. Digital and blended interventions such as Fex-Can and Fex-Can 2.0 have shown that structured psychoeducational content, coupled with opportunities for reflection and support, can be acceptable and useful for survivors [13,14]. EDUSEXONCO is conceptually aligned with this body of work by combining evidence-informed education with multidisciplinary counseling and a supportive group environment to facilitate communication, normalize sexual concerns, and address survivorship-related changes in body image and intimacy. This approach also aligns with frameworks from the WHO and the WAS, which position sexual health as an integral component of health and a human right within survivorship care [17,19]. In Latin American contexts where stigma and sociocultural norms may discourage disclosure, group-based formats may offer a particularly valuable space for peer learning and validation; this feasibility study will help clarify how such delivery functions in the Colombian setting.

### Strengths and Limitations

Strengths of this protocol include the mixed methods design, the participant-centered co-design process, and the manualized intervention structure (facilitator guides, checklists, and fidelity tools) that supports reproducibility. The use of validated instruments (FSFI and EORTC QLQ-C30) enables comparability with prior survivorship research and provides a structured approach to capturing patient-reported outcomes. Qualitative methods will complement questionnaire data by exploring survivors’ lived experiences and perceived relevance of the content and the contextual factors influencing participation.

As a single-center feasibility study using a single-group pretest-posttest design and short follow-up, this study is not intended to provide definitive causal inference or effectiveness estimates. Potential limitations include self-selection and social desirability bias, which may affect acceptability assessments and self-reported outcomes. Feasibility challenges such as scheduling constraints and sustained participation may arise; therefore, prespecified contingency options (eg, shorter session blocks or optional hybrid delivery) are included to support adherence while preserving core intervention components.

### Future Directions

Building on this feasibility study, the next phase of EDUSEXONCO will be designed to evaluate effectiveness and implementation outcomes using a multicenter approach with larger samples and longer follow-up to assess durability and scalability. Hybrid delivery models (combining in-person and digital modules) may be explored to improve accessibility and continuity, particularly for participants in remote or resource-limited settings. Standardized facilitator training and fidelity monitoring will remain essential to ensure consistency across sites. Future studies should also incorporate economic evaluations to assess cost-effectiveness and sustainability. If feasible, the intervention framework could be adapted and tested in other clinical populations affected by sexual health concerns, contributing to a broader biopsychosocial rehabilitation model.

The findings will inform refinement of the EDUSEXONCO curriculum and delivery format (including prespecified contingency options) and will provide context-specific implementation knowledge to support integration of sexual health education into survivorship care pathways in low- and middle-income settings. Results from this feasibility study will also guide the design of a subsequent comparative, multisite study with longer follow-up to evaluate effectiveness and scalability.

## Supplementary material

10.2196/80567 Multimedia Appendix 1 Operational screening checklist.

10.2196/80567 Multimedia Appendix 2 Intervention schedule (per cohort).

10.2196/80567 Multimedia Appendix 3 Detailed topic-by-level syllabus (TIDieR [Template for Intervention Description and Replication] compatible).

10.2196/80567 Multimedia Appendix 4 Example of semistructured group interview guide (sexual health physician session).

## Abbreviations

BETTER: bring up, explain, tell, time, education, record
EORTC QLQ-C30: European Organisation for Research and Treatment of Cancer Quality of Life Questionnaire Core 30
FSFI: Female Sexual Function Index
FVL: Fundación Valle del Lili
PLISSIT: permission, limited information, specific suggestion, intensive therapy
REDCap: Research Electronic Data Capture
WAS: World Association for Sexual Health
WHO: World Health Organization
