# Supplementary material for: Educational Intervention to Improve Sexual Health and Quality of Life in Survivors of Breast and Gynecological Cancer: Protocol for a Mixed Methods Feasibility Study
Source: JMIR Res Protoc. 2026 Feb 27;15:e80567. doi: 10.2196/80567 (PMC12954703; doi:10.2196/80567)
Supplement: Multimedia Appendix 1 [file resprot-v15-e80567-s001.docx]

**Appendix 1. Operational screening checklist**

| # | Item | Criterion (Pass) | How to verify (source) | Value / Notes | Eligible? |
| --- | --- | --- | --- | --- | --- |
| 1 | Age | ≥18 years | EMR demographics; ID card if needed |  | ☐ |
| 2 | Cancer type | Breast or gynecologic (cervical/uterine/vaginal/vulvar/ovarian) | EMR problem list / oncology notes |  | ☐ |
| 3 | Primary treatment completion | ≥24 months before enrollment | EMR oncology summary; last treatment date | Date: ____ | ☐ |
| 4 | Disease status | Disease-free or stable; no urgent treatment planned | Most recent clinic note; oncologist confirmation |  | ☐ |
| 5 | Follow-up site | Active follow-up at Fundación Valle del Lili | Upcoming appointment / EMR follow-up plan |  | ☐ |
| 6 | Language | Spanish-speaking; can complete procedures | Screener judgment during contact |  | ☐ |
| 7 | Attendance feasibility | Able to attend 3 sessions (in-person or hybrid) | Participant confirmation; preferred schedule |  | ☐ |
| 8 | Cognitive capacity | Adequate to consent/participate (or Mini-Cog ≥3 if uncertainty) | Clinician judgment; Mini-Cog result if used |  | ☐ |
| 9 | Medical/ psychiatric stability | No instability that compromises safe participation (e.g., uncontrolled pain crisis; acute severe depression with suicidality; psychosis) | Treating clinician note; participant report |  | ☐ |
| 10 | Conflicting trials | Not enrolled in another sexual-health behavioral study | Participant self-report; research office check if applicable |  | ☐ |

EMR: Electronic Medical Record; ID: Identification.

**Eligibility outcome:** ☐ Eligible ☐ Not eligible
If Not eligible, reason(s): __________________________________________________________________

**Consent obtained (Spanish):** ☐ Yes (date: ________) ☐ No

Recruitment slip present**:** ☐ Yes ☐ No

**Transport needed:** ☐ No ☐ Yes

Arranged by study grant**:** ☐ Booked ☐ Pending (date/time/route): _____________

**Session scheduling** notes: __________________________________________________________________
**Accommodations (e.g., mobility):** ______________________________

**Data protection note:** store this checklist without direct identifiers; keep linkage file (ID -name) encrypted and separate.
