# Supplementary material for: Educational Intervention to Improve Sexual Health and Quality of Life in Survivors of Breast and Gynecological Cancer: Protocol for a Mixed Methods Feasibility Study
Source: JMIR Res Protoc. 2026 Feb 27;15:e80567. doi: 10.2196/80567 (PMC12954703; doi:10.2196/80567)
Supplement: Multimedia Appendix 2 [file resprot-v15-e80567-s002.docx]

**Appendix 2. Intervention Schedule (per cohort)**

**Format:** three sequential sessions (~120 min each) delivered ~weekly.

| Band | Time | Component | Description | Lead(s) | Records/Outputs |
| --- | --- | --- | --- | --- | --- |
| Transport | 7:30–8:00 | Institutional transport (if needed) | Arrival coordination and logistics support | Coordination | Transport log |
| Reception | 8:00–8:30 | Reception & information | Welcome, confidentiality rules, inclusive language, opt‑out option | Principal Investigator / Psychology | Attendance list, context note |
| Questionnaires Session 1 | 8:30–9:00 | Pre‑intervention FSFI + EORTC QLQ‑C30 | Self‑administered (paper/REDCap) | Coordination / Data | Completed questionnaires |
| Theoretical–practical Session 1 | 9:00–10:30 | Medical–sexual content by level | See syllabus (Appendix 3); use of 3D models and materials | Sexology / Gyn Onc / Internist | Fidelity checklist; slide deck version |
| Break | 10:30–11:00 | Coffee break | — | — | — |
| Theoretical–practical Session 2 | 11:00–12:30 | Socio‑psychological content by level | See syllabus (Appendix 3) | Psychology / Social Work | Fidelity checklist |
| Lunch | 12:30–13:30 | Institutional lunch | Rest and socialization | Coordination | — |
| Group interview | 13:30–14:30 | Semi‑structured group interview (6–8 participants) | Experiences, barriers/facilitators, peer learning | Psychology / Social Work (+ physician support) | Audio + field notes; reflective memo |
| Individual counseling | 14:30–16:30 | 1:1 counseling (optional, 30’ each) | According to identified needs | Sexology / Psychology / Social Work | Themes and referrals (no clinical charting) |
| Questionnaires Session 3 | 16:30–17:30 | Post‑intervention FSFI + EORTC QLQ‑C30 | Self‑administered | Coordination / Data | Completed questionnaires |
| Closing | 17:30–18:00 | Closing & next appointment coordination | Key points summary and logistics | Coordination | Net Promoter Score 0–10; anonymous diaries |
| Transport | 18:00–18:30 | Exit transport (if needed) | Per grant/project policy | Coordination | Transport log |

EORTC QLQ-C30: European Organisation for Research and Treatment of Cancer Quality of Life Questionnaire – Core 30; FSFI: Female Sexual Function Index; Gyn Onc (Gynecologic Oncologist).

**Session-specific additions**

- **Session 1 (Baseline):** Before Block B, administer **FSFI** and **EORTC QLQ-C30** (paper or REDCap).
- **Session 3 (Post):** After Block H, administer **FSFI** and **EORTC QLQ-C30**. Offer brief **individual counseling** (10–20 min/participant as needed) with Sexual-health physician/sexologist/Psychologist/Social Worker in parallel room; document only themes and referrals (no clinical charting).
- **Transport/refreshments:** arranged per policy; companions may attend Blocks B–F (not G or individual counseling).
