# Supplementary material for: Educational Intervention to Improve Sexual Health and Quality of Life in Survivors of Breast and Gynecological Cancer: Protocol for a Mixed Methods Feasibility Study
Source: JMIR Res Protoc. 2026 Feb 27;15:e80567. doi: 10.2196/80567 (PMC12954703; doi:10.2196/80567)
Supplement: Multimedia Appendix 3 [file resprot-v15-e80567-s003.docx]

**Appendix 3. Detailed Topic-by-Level Syllabus (TIDieR-compatible)**

Theoretical-practical workshops: These sessions are delivered in large groups and integrate both medical-sexual and socio-psychological content. The workshops are organized in three progressive levels - basic, intermediate, and advanced - to promote a gradual and meaningful learning process. The sessions provide education and practical techniques to positively impact participants’ sexual health, body image, and quality of life

**Structure:** Each level contains a medical-sexual strand and a socio-psychological strand. All items use inclusive, trauma-informed facilitation.

**Level 1 – Basic**

| Strand | Learning objectives (at end of session, participants will…) | Key content | Brief practice | Materials | Lead(s) | Take-home task | Fidelity markers | Qualitative prompts |
| --- | --- | --- | --- | --- | --- | --- | --- | --- |
| Medical–Sexual | 1) Describe post-cancer sexual anatomy/response; 2) Identify ≥2 treatment-related changes (e.g., lubrication, pain); 3) Name ≥2 self-care strategies | Genital & pelvic anatomy refresher; sexual response beyond penetration; common treatment effects (radiotherapy/ chemo/ surgery); lubricants & moisturizers; safety basics | “Anatomy tour” with 3D model; lubricant types matching game; 3-step “comfort plan” | Slides, 3D models, sample lubricants/ moisturizers (non-use demo), handout (reading level B1–B2) | SH-phys / Gyn Onc | Fill “My Comfort Plan” and bring 1 question next session | ≥80% key items covered; demo shown; handouts issued | “What changed in your body since treatment?” “What feels safer/more comfortable now?” |
| Socio-Psych | 1) Name personal fears/ expectations; 2) Identify ≥1 sociocultural barrier; 3) Practice a basic communication script | Myths vs facts; stigma & shame; communicating needs/ preferences; boundaries & consent; rights-based framing | 5-line “Ask-Acknowledge-Agree” script; boundary sentence stems | Scripts card, myth/ fact cards | Psychologist / Social Worker | Practice script with trusted person (or journaling if no partner) | Role-play completed; R/A done | “What messages did you receive about sexuality? “What would feel safe to try?” |

Gyn Onc: Gynecologic Oncologist; R/A: Reflection/Assessment; SH-phys: Sexual-health physician/sexologist.

**Level 2 –** **Intermediate**

| Strand | Learning objectives | Key content | Brief practice | Materials | Lead(s) | Take-home task | Fidelity markers | Qualitative prompts |
| --- | --- | --- | --- | --- | --- | --- | --- | --- |
| Medical–Sexual | 1) Define pleasure beyond intercourse; 2) Identify ≥2 autoerotic practices aligned with comfort; 3) Recognize options for dyspareunia | Pleasure mapping; sensate focus basics; positions and pacing; device overview (vibrators, dilators: indications, hygiene); pain-fear cycle | Sensate-focus step 1 (solo); paced breathing; neutral-to-pleasant ladder | Device pictures (no explicit use), position guide sheet | SH-phys / PFPT | 10-min body-scan; note one “pleasant neutral” activity | Content checklist complete; safety emphasized | “What feels pleasurable/ neutral?” “What helps when pain appears?” |
| Socio-Psych | 1) Reframe body image with self-compassion; 2) Identify supports; 3) Plan one social reintegration step | Body image after cancer; self-compassion micro-skills; social re-entry; negotiating pace with partner/family | 7-minute self-compassion break; “support map” | Worksheets; small group flipchart | Psychologist / Social Worker | Choose one gentle reintegration action and journal response | Worksheet completion; peer share done | “How do you speak to yourself?” “Who/what supports you?” |

PFPT = Pelvic-floor physical therapist; SH-phys = Sexual-health physician/sexologist.

**Level 3 –** **Advanced**

| Strand | Learning objectives | Key content | Brief practice | Materials | Lead(s) | Take-home task | Fidelity markers | Qualitative prompts |
| --- | --- | --- | --- | --- | --- | --- | --- | --- |
| Medical–Sexual | 1) Apply nutrition & pelvic-floor strategies to sexual comfort; 2) Select suitable lubricants/devices; 3) Draft a personal activation plan | Energy & mood-supportive nutrition; PF basics (relax/contract; down-training for pain); lubricant/device selection; when to seek specialist care | PF “down-training” + diaphragmatic breathing; lubricant/device selection checklist | PF cue cards; selection checklist | PFPT / Nutritionist / SH-phys | 10–15 min/day PF + breathing; update activation plan | Demonstrations completed; plan drafted | “What strengthens your confidence?” “What’s your first small step?” |
| Socio-Psych | 1) Use emotion regulation under stress; 2) Practice yoga/ relaxation tailored to limitations; 3) Consolidate support plan | Emotion regulation micro-skills; paced yoga/ relax; relapse-prevention for low mood/anxiety; community resources | 10-minute gentle sequence; 4-7-8 breathing; crisis card | Mats/chairs; breathing card; resource list | Yoga Instructor / Psychologist | 3-day log (breathing/yoga + mood note) | Sequence taught as planned; safety screening done | “What helps you re-center?” “How will you sustain gains?” |

PF: Pelvic-floor; PFPT: Pelvic-floor physical therapist; SH-phys: Sexual-health physician/sexologist.

 **Session 1 (pre):** FSFI, EORTC QLQ-C30.

 **All sessions (post-block H):** Anonymous diary (acceptability), 0–10 recommendation item (Net Promoter Score calc), short facilitator memo (fidelity/feasibility).

 **Session 3 (post):** FSFI, EORTC QLQ-C30; record any **co-interventions** disclosed (e.g., new topical estrogen, Pelvic-floor therapy).
