# Supplementary material for: Educational Intervention to Improve Sexual Health and Quality of Life in Survivors of Breast and Gynecological Cancer: Protocol for a Mixed Methods Feasibility Study
Source: JMIR Res Protoc. 2026 Feb 27;15:e80567. doi: 10.2196/80567 (PMC12954703; doi:10.2196/80567)
Supplement: Multimedia Appendix 4 [file resprot-v15-e80567-s004.docx]

**Appendix 4. Example of Semi-Structured Group Interview Guide (Sexual-Health Physician Session)**

**1. Overall structure of EDUSEXONCO group interviews**

**Type of activity**

Semi-structured group interviews / facilitated group discussions with women who are survivors of gynecologic cancer, conducted in a workshop format.

**Facilitating professionals**

- Sexual-health physician (internist with training in sexual health)
- Gynecologic oncologist
- Psychiatrist

**Common phases across all sessions**

1. **Opening:** Welcome, brief explanation of the purpose of the session, and reminder of group agreements (confidentiality, respect, voluntary participation, right to pause or not answer).
2. **Initial broad prompt:** An open-ended question to invite participants to share their perceptions and questions (eg, “How did you feel during today’s session?”, “What doubts do you have?”, “What did you understand or what stayed with you?”).
3. **Thematic exploration:** Progression through pre-defined thematic axes, using follow-up prompts (probes) tailored to what participants bring up spontaneously.
4. **Closing:** Summary of key ideas, space for final questions, and acknowledgment of participants’ contributions and emotional effort.

**2. Semi-structured guide – Sexual-health session**

This guide focuses on: definitions of sexual health, genital and clitoral anatomy, guilt and moral norms, family narratives, orgasm and pleasure, relationships with partners, and medical/rehabilitative options (eg, hormone therapy, pelvic-floor therapy, sexual aids, dilators).

**2.1. Opening**

**Objective:**
To break the ice and introduce the topic of sexual health and pleasure in a safe, non-judgmental way.

**Core opening question:**

- “I would like to know whether the concept of sexual health is clearer for you after today’s session, and whether this has changed, in any way, how you think about sexual health and sexual pleasure.”

**Sample probes:**

- “What new things did you hear today that you did not know before?”
- “What did you find most surprising?”

**2.2. Previous representations of sexual health and pleasure**

**Objective:**
To explore how participants were originally taught to think about sexual health and what ideas they bring from earlier life stages.

**Guiding questions:**

1. “Before attending these sessions, how had you been taught to see sexual health? Where did you mainly learn about it (eg, family, school, church, television, internet)?”
2. “Were you taught to think about your own pleasure, or mainly about satisfying a partner?”

**Sample probes:**

- “Do you remember any typical phrases from your parents or caregivers about sex, menstruation, or ‘being a good woman’?”
- “When you tried to ask questions about sexual health in the past, was there silence, fear, or shame?”

**2.3. Anatomical knowledge and discovery of the clitoris**

**Objective:**
To make visible the learning about body and genital anatomy, particularly the clitoris and its role in pleasure.

**Guiding questions:**

1. “When we reviewed the anatomy, especially the clitoris, what did you feel or think? Did you imagine it was that large, or did you think it was only the small part visible on the outside?”
2. “How was it for you to connect that anatomical information with your own experiences of pleasure or pain?”

**Sample probes:**

- “Had you ever seen drawings or 3D models of the clitoris and genital area in such detail before?”
- “Did this change anything in how you understand your orgasms or the difficulties you may have had reaching orgasm?”

**2.4. Guilt, religious norms, and the mandate to “satisfy the other”**

**Objective:**
To verbalize the moral and emotional burden many women carry regarding sexual health.

**Guiding questions:**

1. “In your own life stories, how has guilt or the idea that sexual health is ‘bad’, ‘dirty’, or ‘forbidden’ been present?”
2. “Several of you have mentioned being raised to satisfy your partner’s needs. How have you experienced that expectation? What consequences has it had for you?”

**Sample probes:**

- “Have you ever felt afraid to say what you like sexually because of comments such as ‘Where did you learn that?’ or ‘Who taught you that?’”
- “How do you feel today when you think about that idea of always having to satisfy the other person?”

**2.5. Effects of oncologic and hormonal treatments on** sexual health

**Objective:**
To connect physical and hormonal changes after cancer treatment with lived sexual experiences.

**Guiding questions:**

1. “For those who have undergone surgeries such as hysterectomy or removal of the ovaries, what changes have you noticed in your desire, lubrication, or ability to reach orgasm?”
2. “How has your experience been with symptoms such as vaginal dryness, hot flashes, fatigue, or pain during sexual activity?”

**Sample probes:**

- “Has anyone received hormone therapy (systemic or local)? How was it explained to you?”
- “Do you feel that your doctors have adequately informed you about how treatments could affect your sexual life?”

**2.6. Resources and therapies: pelvic-floor work, dilators, lubricants, devices**

**Objective:**
To explain therapeutic options and reduce stigma around medical devices and sexual aids.

**Guiding questions:**

1. “Before today, what did you know about pelvic-floor exercises, Kegel balls, vaginal dilators, laser or plasma treatments, or lubricants?”
2. “How do you feel now after learning that many devices that are seen as ‘sexual toys’ actually originated as medical tools to strengthen the vagina, support rehabilitation, and help with pleasure?”

**Sample probes:**

- “What fears or prejudices have you had regarding these devices or treatments?”
- “Can you imagine yourself using any of these resources? What would you need in order to feel comfortable trying them (eg, more information, partner support, medical guidance)?”

**2.7. Session closing**

**Objective:**
To reinforce the central message that women have a right to sexual pleasure and that pain or discomfort should not be considered “normal” or inevitable.

**Guiding closing questions:**

1. “After everything we have discussed today, what message do you take with you about your right to sexual pleasure and about not normalizing uncomfortable or painful situations?”
2. “What is the main idea you would like to remember from this session when you are at home, with your own body or with a partner?”
